# Molecular Characterization and Clinical Relevance of RNA Binding Proteins in Colorectal Cancer

**DOI:** 10.3389/fgene.2020.580149

**Published:** 2020-10-16

**Authors:** Zhen Zhang, Ling Wang, Quan Wang, Mengmeng Zhang, Bo Wang, Kewei Jiang, Yingjiang Ye, Shan Wang, Zhanlong Shen

**Affiliations:** ^1^Department of Gastroenterological Surgery, Peking University People’s Hospital, Beijing, China; ^2^Laboratory of Surgical Oncology, Beijing Key Laboratory of Colorectal Cancer Diagnosis and Treatment Research, Peking University People’s Hospital, Beijing, China; ^3^Key Laboratory of Carcinogenesis and Translational Research (Ministry of Education), Hepatopancreatobiliary Surgery Department I, Peking University Cancer Hospital and Institute, Beijing, China

**Keywords:** colorectal cancer, RNA binding protein, prognostic model, transcriptomics, TCGA

## Abstract

Abnormal expression of RNA binding proteins (RBPs) has been reported across various cancers. However, the potential role of RBPs in colorectal cancer (CRC) remains unclear. In this study, we performed a systematic bioinformatics analysis of RBPs in CRC. We downloaded CRC data from The Cancer Genome Atlas (TCGA) database. Our analysis identified 242 differentially expressed RBPs between tumor and normal tissues, including 200 upregulated and 42 downregulated RBPs. Next, we found eight RBPs (RRS1, PABPC1L, TERT, SMAD6, UPF3B, RP9, NOL3, and PTRH1) related to the prognoses of CRC patients. Among these eight prognosis-related RBPs, four RBPs (NOL3, PTRH1, UPF3B, and SMAD6) were selected to construct a prognostic risk score model. Furthermore, our results indicated that the prognostic risk score model accurately predicted the prognosis of CRC patients [area under the receiver operating characteristic curve (AUC)for 3- and 5-year overall survival (OS) and was 0.645 and 0.672, respectively]. Furthermore, we developed a nomogram based on a prognostic risk score model. The nomogram was able to demonstrate the wonderful performance in predicting 3- and 5-year OS. Additionally, we validated the clinical value of four risk genes in the prognostic risk score model and identified that these risk genes were associated with tumorigenesis, lymph node metastasis, distant metastasis, clinical stage, and prognosis. Finally, we used the TIMER and Human Protein Atlas (HPA)database to validate the expression of four risk genes at the transcriptional and translational levels, respectively, and used a clinical cohort to validate the roles of NOL3 and UPF3B in predicting the prognosis of CRC patients. In summary, our study demonstrated that RBPs have an effect on CRC tumor progression and might be potential prognostic biomarkers for CRC patients.

## Introduction

Colorectal cancer (CRC) is one of the most common cancers of the gastrointestinal tract. It is the third leading cause of cancer-related death worldwide (Siegel et al., 2020). Although surgical and adjuvant therapies have improved, the 5-year overall survival (OS) rate of CRC patients ranges from 90 to 10% (Van Cutsem et al., 2014). The poor prognosis of CRC is primarily due to tumor distant metastasis and recurrence (Roth et al., 2010; Kow, 2019). Therefore, understanding the mechanisms that lead to CRC initiation and progression is necessary for diagnosis, therapeutic interventions, and prognostic prediction.

RNA binding proteins (RBPs) are a class of proteins involved in splicing, modifications, transport, localization, stability, degradation, and translation of RNAs (Mitchell and Parker, 2014; Perron et al., 2018). In fact, more than 1,500 human RBPs have been validated by high-throughput screens and experiments (Gerstberger et al., 2014; Neelamraju et al., 2015). RBPs play vital roles in several essential cellular processes by interacting with their target RNAs (Moore, 2005). The target RNAs of RBPs are diverse and include microRNAs, transfer RNAs (tRNAs), small interfering RNA, small nucleolar RNAs, and small nuclear RNAs (Hentze et al., 2018). Abnormally expressed RBPs regulate the expression and function of oncogenes and tumor-suppressor genes via post-transcriptional regulatory mechanisms across various cancers. For example, aberrant hnRNPM expression promoted breast cancer metastasis by controlling CD44 splice isoform switching during epithelial–mesenchymal transition (EMT) (Xu et al., 2014). In melanomas, CPEB4 increased the translation of melanoma drivers MITF and RAB72A, which helps promote tumor proliferation (Pérez-Guijarro et al., 2016). In hepatocellular carcinoma, HuR/methyl-HuR and AUF1 modulate MAT1A and MAT2A expression through post-translational regulation of their messenger RNAs (mRNAs), thus impacting tumor progression (Vázquez-Chantada et al., 2010). In CRC, overexpression of IMP-1 increased proliferation by directly binding to and stabilizing c-Myc (Mongroo et al., 2011).

With the rapid development of high-throughput sequencing, researchers have have been able to perform a systematic functional analysis of RBPs using high-throughput bioinformatics profiling. Recent bioinformatics studies have implied that RBPs can predict the prognosis of different cancers, such as breast cancer, lung adenocarcinoma, glioma, hepatocellular carcinoma, and leukemia (Li et al., 2019a, b, 2020; Saha et al., 2019; Wang K. et al., 2019,Wang Z. et al., 2020). In this study, we downloaded CRC data from the Cancer Genome Atlas (TCGA) database. Next, we selected differentially expressed RBPs to perform Gene Ontology (GO) and Kyoto Encyclopedia of Genes and Genomes (KEGG) pathway analyses. Furthermore, we identified prognostic RBPs that enable the construction of a prognostic risk score model. Furthermore, we built a nomogram based on the prognostic risk score model. Finally, we explored the clinical value of these risk genes. Our study detected that several RBPs are involved in CRC, which might be used to predict the prognosis of CRC patients and inhibit tumor progression in the future.

## Materials and Methods

### Data Acquisition

RNA sequencing data and the corresponding clinical data were downloaded from the TCGA database^1^. Overall, 568 CRC patients were used to analyze the differentially expressed RBPs in the TCGA database. Then, 489 CRC patients were selected for clinical analyses, as these patients had complete clinical information, including primary tumor, lymph node metastasis, distant metastasis, clinical stage, and follow-up for at least 1 month. Additionally, we downloaded RNA-seq data of the GSE29623 cohort from the Gene Expression Omnibus (GEO) database^2^. In total, 1,542 RBPs were included in our study (Gerstberger et al., 2014; Neelamraju et al., 2015).

### Clinical Samples and Immunohistochemistry Staining

In total, tumor and normal tissues of 44 CRC patients were obtained from the Peking University People’s Hospital. All tissues were histopathologically confirmed by pathologists. This study was granted approval by the ethics committee of Peking University People’s Hospital. Immunohistochemistry (IHC)staining was performed according to prior published protocols (Zhang et al., 2019). Briefly, the staining index scores were assigned as follows: staining intensity (negative: 0; weak: 1; moderate: 2; strong: 3) and positive staining (<5%: 0; 5–25%: 1; 26–50%: 2; 51–75%: 3; > 75%: 4). The staining index scores were calculated by multiplying the staining intensity score by the positive staining score, which ranged from 0 to 12. The antibodies used in this study included anti-NOL3 (Proteintech, United States) and anti-UPF3B (Proteintech, United States).

### Functional Enrichment Analysis of Differentially Expressed RNA Binding Proteins

The GO and KEGG pathway analyses were performed to analyze the biological functions of these differentially expressed RBPs. The GO terms include biological process (BP), cellular component (CC), and molecular function (MF).

### Building a Prognostic Model

A univariate Cox regression analysis was performed to identify prognostic RBPs. Subsequently, a multivariable Cox regression analysis was carried out to construct a prognostic risk score model. Furthermore, the risk score was calculated as follows:

$$riskscore=\sum_{i=1}^{n}coefficieent{\left(i\right)}^{*}expression\left(i\right),$$

where coefficient(i) and expression(i) represent the regression coefficient and expression levels of selected genes in the prognostic risk score model, respectively. The time-dependent receiver operating characteristic (ROC) analysis was used to assess the prognostic ability of the prognostic risk score model. Then, a nomogram was built to predict the OS of CRC patients. The calibration plot and concordance index (C−index) were used to evaluate the performance of the nomogram.

### External Validation of the Expression and Genetic Alterations of the Risk Genes

The TIMER database^3^ and the Human Protein Atlas (HPA) database^4^ were utilized to explore and validate gene expression at the transcriptional and translational level, respectively. The cBioportal for Cancer Genomics^5^ was used to identify each genetic alteration, which included four CRC studies.

### Statistical Analyses

Statistical analyses were performed using the R software v3.5.1. Kaplan–Meier analysis was used to construct survival curves, and the log-rank test was utilized to assess the significance of differences. *T*-test and Wilcoxon signed-rank test were used to explore quantitative variables. *P* < 0.05 represents statistical significance.

## Results

### Identifying Differentially Expressed RNA Binding Proteins in Colorectal Cancer

**FIGURE 1:**
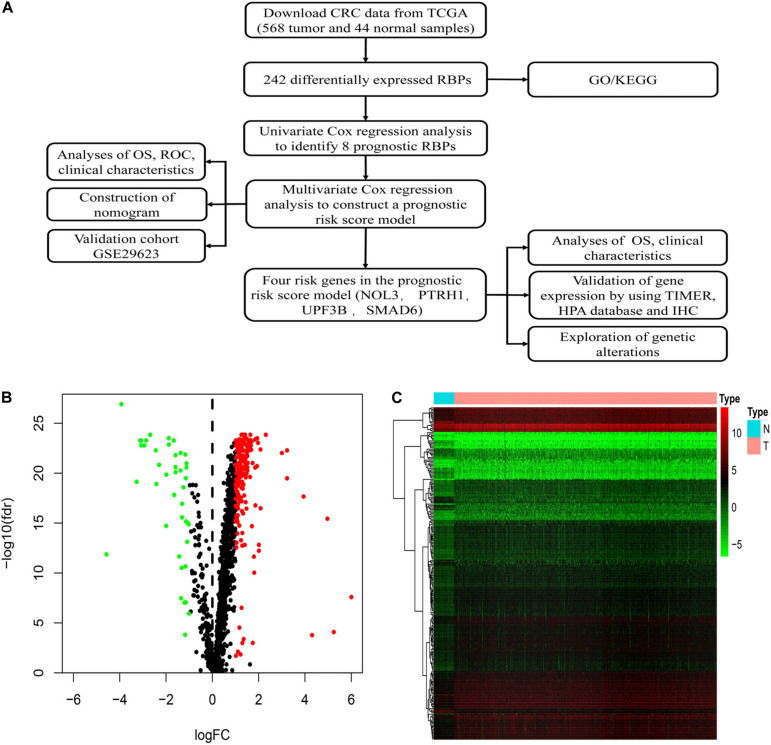
Identification of differentially expressed RNA binding proteins (RBPs) in colorectal cancer (CRC). **(A)** Flowchart of our methods. **(B)** Volcano plots of differentially expressed RBPs in CRC. **(C)** Heatmap plots of differentially expressed RBPs in CRC.

Figure 1A shows detailed designs of the study. We downloaded transcriptomic files of CRC from the TCGA database encompassing 568 tumors and 44 normal samples. The Wilcoxon signed-rank test was applied to identify significantly differentially expressed RBPs. The R package Limma was used to identify differentially expressed RBPs according to the following parameters: | log_2_FC| > 1 and false discovery rate (FDR) < 0.05. Among the 1,542 RBPs, we detected 242 differentially expressed RBPs, including 200 upregulated and 42 downregulated RBPs between tumor and normal tissues (Figures 1B,C).

### Functional Enrichment Analysis of Differentially Expressed RNA Binding Proteins in Colorectal Cancer

**FIGURE 2:**
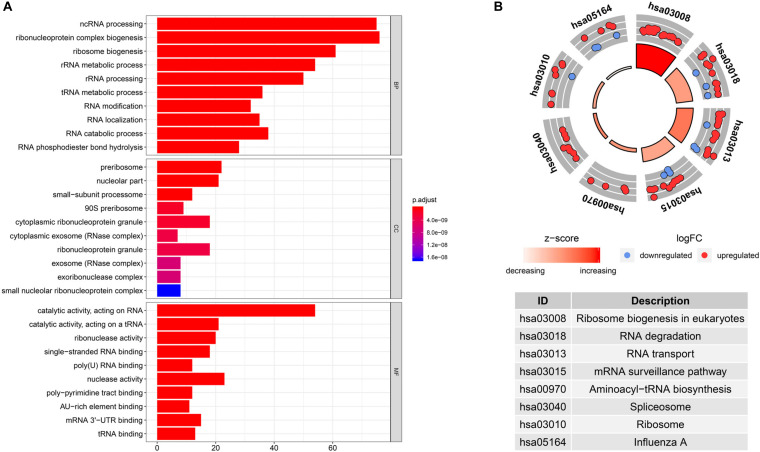
Gene Ontology (GO) enrichment and Kyoto Encyclopedia of Genes and Genomes (KEGG) pathway analysis of differentially expressed RBPs in CRC. **(A)** GO terms of differentially expressed RBPs. **(B)** KEGG pathways of differentially expressed RBPs.

To investigate the biological significance of RBPs in CRC, we performed GO and KEGG pathway analyses of 242 differentially expressed RBPs using the R package clusterProfiler. We displayed the top 10 significantly enriched GO terms (Figure 2A). Results revealed that these differentially expressed RBPs were significantly enriched in biological processes such as non-coding RNA processing, ribonucleoprotein complex biogenesis, ribosome biogenesis, and ribosomal RNA metabolism. They were mainly located in the preribosome, nucleolar part, small-subunit processome, 90S preribosome, and cytoplasmic ribonucleoprotein granule. They were found to participate in various molecular functions, including catalytic activity (acting on RNA), catalytic activity (acting on a tRNA), ribonuclease activity, single-stranded RNA binding, and poly(U) RNA binding. For KEGG pathway analysis, we found that these differentially expressed RBPs were mainly associated with ribosome biogenesis in eukaryotes, RNA degradation, RNA transport, mRNA surveillance pathway, and aminoacyl-tRNA biosynthesis (Figure 2B).

### Identifying Prognostic RNA Binding Proteins in Colorectal Cancer

**FIGURE 3:**
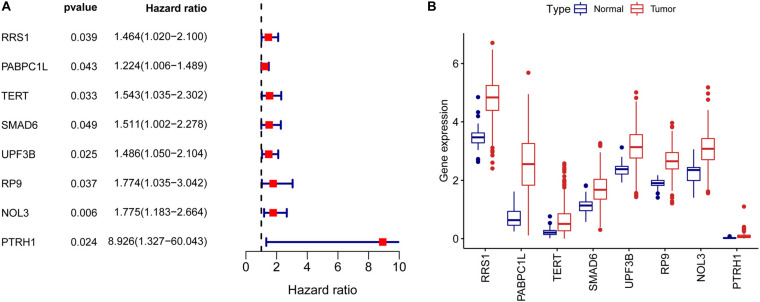
Univariate Cox regression analysis to identify prognostic RBPs in CRC. **(A)** Forrest plot of univariate Cox regression analyses in CRC. **(B)** mRNA expression of eight prognostic RBPs in CRC.

To assess the prognostic significance of RBPs in CRC, we performed a univariate Cox regression analysis of 242 differentially expressed RBPs. We detected eight RBPs (RRS1, PABPC1L, TERT, SMAD6, UPF3B, RP9, NOL3, and PTRH1) that were related to the prognosis of CRC patients (*p* < 0.05, Figure 3A). Also, all these eight prognostic RBPs were protective factors because CRC patients with high expression of these RBPs had poor prognosis. The expression of eight prognostic RBPs in tumor and normal tissues of CRC patients are shown in Figure 3B.

### Constructing a Prognostic Risk Score Model in Colorectal Cancer

**FIGURE 4:**
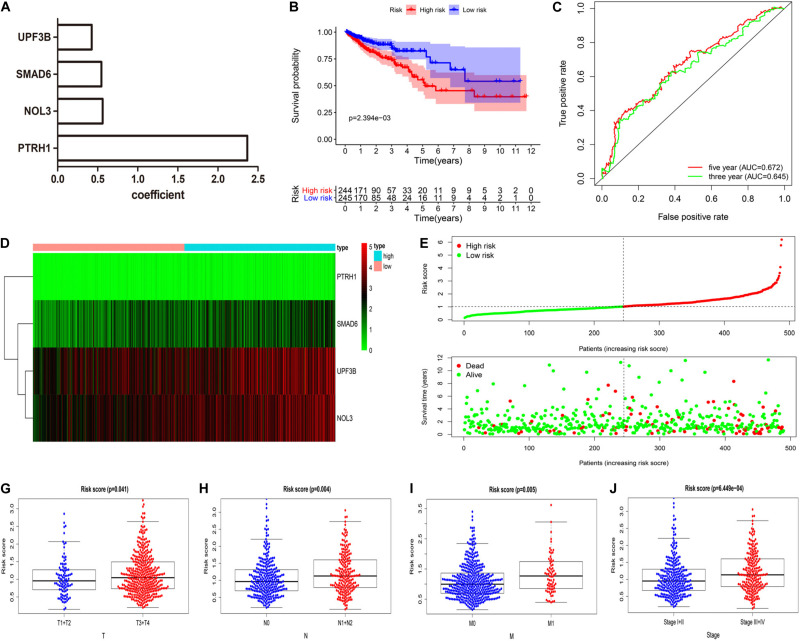
Four RBP-related gene model to predict overall survival (OS) of CRC patients. **(A)** Coefficient of four risk genes identified by multivariate Cox regression analysis. **(B)** OS curve for high-risk and low-risk groups in the prognostic risk score model. **(C)** Time-dependent receiver operating characteristic (ROC) analysis of the prognostic risk score model.**(D–F)** Heat map of mRNA expression **(D)**, distribution of risk score **(E)**, and survival status **(F)** of patients in high-risk and low-risk groups. **(G–J)** Relationships between risk score and T **(G)**, N **(H)**, M **(I)**, and clinical stage **(J)**. (T, primary tumor; N, lymph node metastasis; M, distant metastasis).

We constructed the optimum prognostic risk score model for prediction of OS of CRC patients by using multivariate Cox regression analysis. The identified eight prognostic RBPs were used to construct the prognostic risk score model. Among the eight prognostic RBPs, we identified UPF3B, SMAD6, NOL3, and PTRH1 as risk genes in the prognostic risk score model. Furthermore, coefficients of four risk genes are shown in Figure 4A. We calculated the risk scores using regression coefficient and expression levels of the risk genes according to this equation: risk score = 0.4257 ^∗^ expression (UPF3B) + 0.5463 ^∗^ expression (SMAD6) + 0.562 ^∗^ expression (NOL3) + 2.368 ^∗^ expression (PTRH1). Then, all CRC patients were divided into either high-risk or low-risk groups according to the risk scores. We demonstrated that patients in the high-risk group had a shorter OS time compared with those in the low-risk group (Figure 4B). We measured the prognostic ability of the risk score model through the use of an ROC analysis, which was conducted using the R package survivalROC. Also, our results indicated that the area under the ROC curve for 3- and 5-year OS was 0.645 and 0.672, respectively (Figure 4C). Heat map of mRNA expression indicated that all four were upregulated in the high-risk group (Figure 4D). Distribution of risk scores and survival status of patients are shown in Figures 4E,F. In addition, we found a higher percentage of deaths in the high-risk group.

We next assessed whether the four RBP-related gene models can predict the survival prognosis of CRC patients among additional CRC cohorts. We calculated the risk scores using the same formula that was used in the GSE29623 cohort. The results demonstrated that patients in the high-risk group had shorter OS time compared with those in the low-risk group (Supplementary Figures 1A–D), which is consistent with the TCGA CRC cohort. The results demonstrated that the four RBP-related gene models can accurately predict the prognosis of CRC patients.

Additionally, we explored the relationship between risk scores and clinical features of CRC patients. These results implied that risk scores were significantly higher in CRC patients that have deeper tumor infiltration (Figure 4G), distant metastasis (Figure 4H), and lymph node metastasis (Figure 4I) and are at a late clinical stage (Figure 4J).

### Building a Nomogram Based on the Prognostic Risk Score Model

**FIGURE 5:**
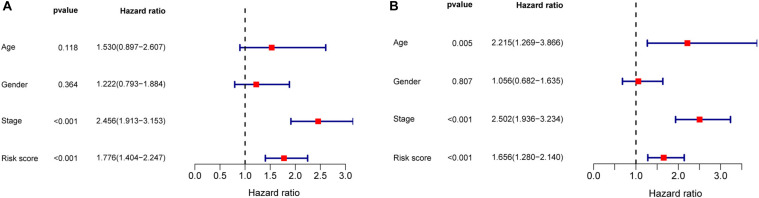
Univariate and multivariate Cox regression analyses in CRC. **(A)** Forrest plot of the univariate Cox regression analyses in CRC. **(B)** Forrest plot of the multivariate Cox regression analyses in CRC.

**FIGURE 6:**
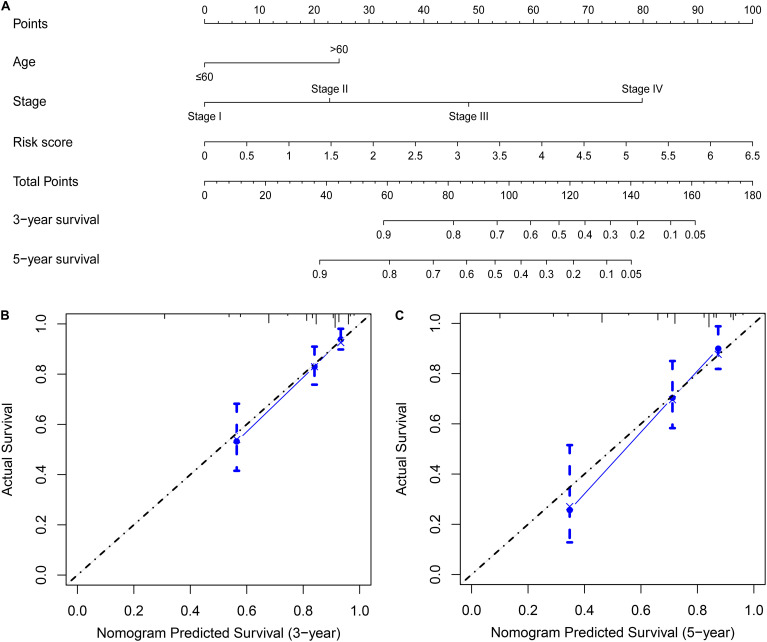
Building a nomogram predicting OS of CRC patients. **(A)** CRC survival nomogram. The calibration plot for predicting patient survival at **(B)** 3 and **(C)** 5 years.

Univariate and multivariate Cox regression analyses demonstrated that age, clinical stage, and the risk scores obtained from the prognostic risk score model were independent prognostic factors of CRC patients (Figures 5A,B). Therefore, based on these three independent prognostic factors, we constructed a nomogram that would be able to predict 3- and 5-year OS of CRC patients using the R package rms (Figure 6A). The calibration plots showed that the probability of predicting 3- or 5-year OS through nomogram agreed with actual observation (Figures 6B,C). Furthermore, the C-index for predicting OS through nomogram was 0.75 (95% CI: 0.69–0.81).

### Validating Clinical Value of the Four Risk Genes in the Prognostic Risk Score Model

**FIGURE 7:**
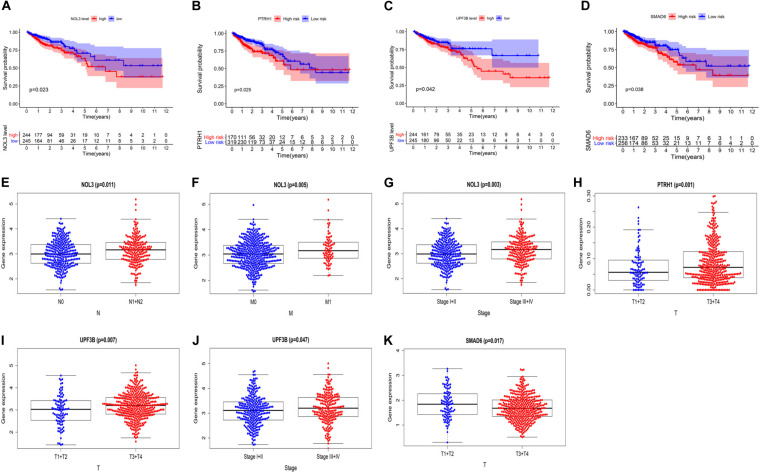
Relationship between the four risk genes expression and clinical features of CRC patients in The Cancer Genome Atlas (TCGA). **(A–D)** Survival curves of NOL3 **(A)**, PTRH1 **(B)**, UPF3B **(C)**, and SMAD6 **(D)**. **(E–G)** Relationships between NOL3 expression and N **(E)**, M **(F)**, and clinical stage **(G)**. **(H)** Relationships between PTRH1 expression and T. **(I,J)** Relationships between UPF3B expression and T **(I)** and clinical stage **(J)**. **(K)** Relationships between SMAD6 expression and T. (T, primary tumor; N, lymph node metastasis; M, distant metastasis).

To further explore the clinical value of these four risk genes, we analyzed the relationship between expression of these genes and clinical features of the CRC patients. The survival curves showed that high expression of NOL3, PTRH1, SMAD6, and UPF3B is associated with poor prognosis of CRC patients (Figures 7A–D). We also found that NOL3 was significantly overexpressed in patients with lymph node metastasis (Figure 7E), distant metastasis (Figure 7F), and at a late clinical stage (Figure 7G). PTRH1 was found to be significantly overexpressed in patients with deeper tumor infiltration (Figure 7H). UPF3B was significantly upregulated in patients with deeper tumor infiltration (Figure 7I), as well as late clinical stage (Figure 7J). However, SMAD6 was downregulated in patients with deeper tumor infiltration (Figure 7K).

### External Validation of the Four Risk Genes

**FIGURE 8:**
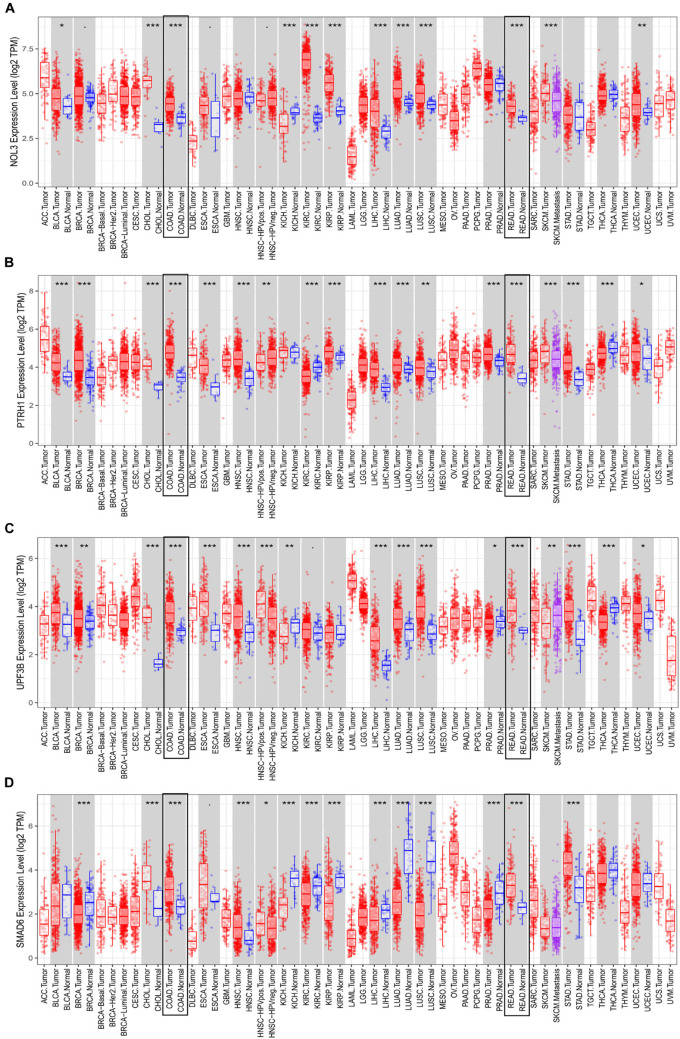
Expression of the four risk genes in multiple cancers. Expression of **(A)** NOL3, **(B)** PTRH1, **(C)** UPF3B, and **(D)** SMAD6 in TIMER. *p < 0.05, **p < 0.01, ***p < 0.001.

Consistent with results from the TCGA database, each of the four risk genes was found to be significantly upregulated in both colon and rectal cancer, according to the TIMER database (Figure 8). Interestingly, we identified that the expression of these four genes is not the same across different cancers. For instance, UPF3B is upregulated in esophageal carcinoma, stomach adenocarcinoma, hepatocellular carcinoma, and breast invasive carcinoma, whereas UPF3B was downregulated in kidney chromophobe, prostate adenocarcinoma, and thyroid carcinoma. Additionally, the expression of UPF3B was not found to be significantly different in pancreatic adenocarcinoma, kidney renal clear cell carcinoma, and kidney renal papillary cell carcinoma.

**FIGURE 9:**
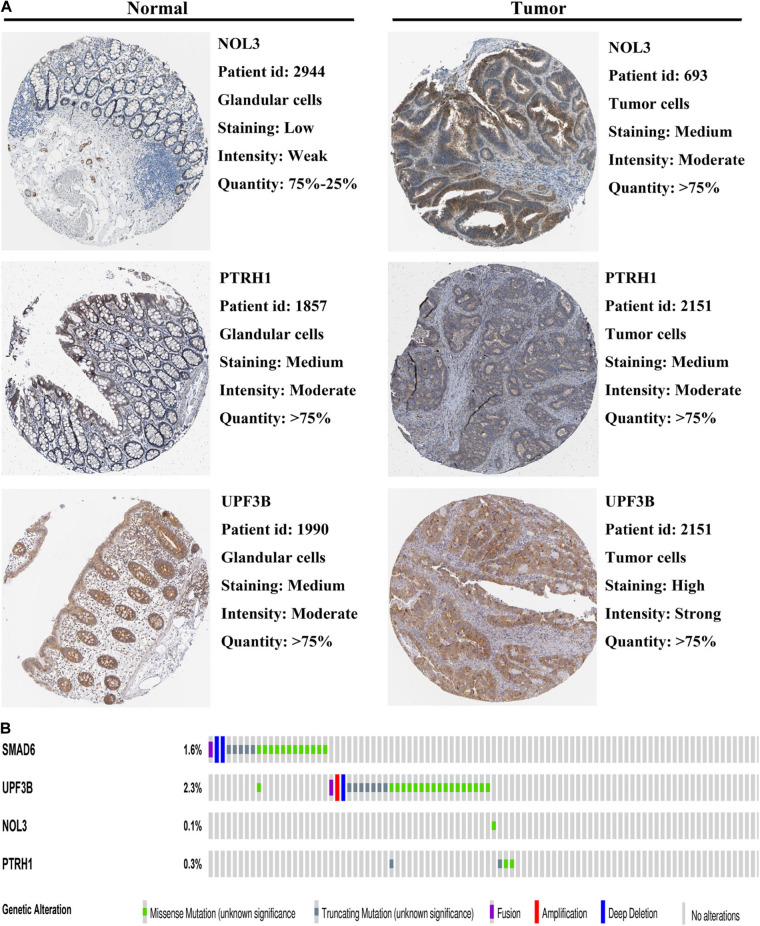
Protein expression and genetic alterations of the four risk genes. **(A)** Representative protein level of the four risk genes in CRC tumor and normal tissues in the Human Protein Atlas database. Data of SMAD6 are not available in this database. **(B)** Genetic alterations of the four risk genes in CRC in the cBioportal for Cancer Genomics.

To further validate the expression of the four risk genes at the translational level, we analyzed the protein expression of these genes in the HPA database. The results indicated that NOL3 and UPF3B were overexpressed in CRC tumor tissues compared with normal tissues (Figure 9A). PTRH1 expression was not significantly different between the CRC tumor tissues and normal tissues (Figure 9A). However, information of SMAD6 levels were not found on the website. In addition, genetic alterations of the four risk genes were found to rarely occur (Figure 9B).

### Validation of the Clinical Significance of NOL3 and UPF3B in Colorectal Cancer Patients by Immunohistochemistry

**FIGURE 10:**
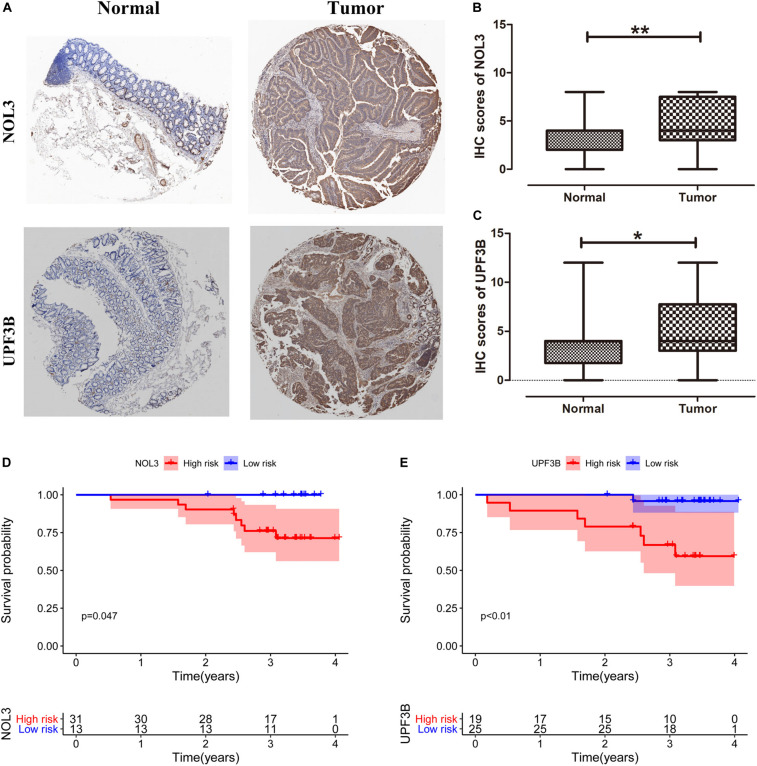
Clinical significance of NOL3 and UPF3B in CRC patients by immunohistochemistry (IHC). **(A)** IHC staining for NOL3 and UPF3B in tumor and normal tissues of CRC patients. **(B,C)** Statistical analysis of NOL3 and UPF3B in tumor and normal tissues of CRC patients, respectively. **(D,E)** Survival curves of NOL3 and UPF3B. *p < 0.05, **p < 0.01.

We obtained 44 pairs of CRC samples from Peking University People’s Hospital to validate protein expression of the two key RBPs (NOL3 and UPF3B) in CRC. Immunohistochemical staining results displayed that NOL3 and UPF3B were upregulated in CRC tumor tissues (Figures 10A–C). In addition, our results showed that overexpression of NOL3 and UPF3B was associated with poor prognosis of CRC patients (Figures 10D,E). These results indicated that NOL3 and UPF3B play vital roles in predicting the prognosis of CRC patients.

## Discussion

Recently, numerous studies have focused on certain characteristics, such as autophagy and metabolic reprogramming, to identify gene signatures that are able to predict the mortality risk of cancer (Wan et al., 2019; Lin et al., 2020; Liu et al., 2020). In this study, we identified four RBP-related genes that were able to predict the OS of CRC patients. First, we detected 242 differentially expressed RBPs from a total of 1,542 RBPs. Then, we performed univariate and multivariable Cox regression analyses and selected four prognosis-related RBPs to construct a prognostic risk score model. Also, results showed that the prognostic risk score model accurately predicted prognosis of CRC patients. Furthermore, we built a nomogram based on independent prognostic factors (including the risk score obtained from the prognostic risk score model, age, and clinical stage). Also, the nomogram performed well in predicting the 3- and 5-year OS. We also validated the clinical value of the four risk genes and found that they were associated with tumorigenesis, lymph node metastasis, distant metastasis, clinical stage, and OS. Finally, we confirmed the vital roles of NOL3 and UPF3B in predicting prognosis of CRC patients using IHC in a clinical cohort.

Based on four prognosis-related RBPs, we constructed a prognostic risk score model to predict OS of CRC patients. Some of these genes were found to be related to tumorigenesis and progression of CRC and other malignancies. NOL3 was strongly upregulated across multiple cancers. In particular, NOL3 was found to be highly expressed in AML and was associated with poor prognosis of AML patients (Carter et al., 2011, 2019; Mak et al., 2014a, b). Overexpression of NOL3 was also related to poor prognosis of nasopharyngeal carcinoma patients (Wu et al., 2013). Studies also found that NOL3 promoted tumorigenesis, metastasis, and chemoresistance in breast cancer, all of which contributed to worse patient prognosis (Medina-Ramirez et al., 2011). NOL3 was a direct target of miR-185 in gastric cancer (Li et al., 2014). Recent discoveries have also identified that upregulation of NOL3 was associated with worse prognosis among CRC patients (Mercier et al., 2008; Tóth et al., 2016), which is consistent with our results. Finally, NOL3 might be modulated by known cancer signaling proteins including Ras (Wu et al., 2010) and HIF-1 (Ao et al., 2012), and the lncRNA PCAT6 (Huang et al., 2019), in CRC.

SMAD6, a member of the SMAD family, negatively modulates the transforming growth factor-β signaling pathway (Jung et al., 2013). SMAD6 is predictive of patient survival in oral squamous cell carcinoma (Mangone et al., 2010). SMAD6 was found to be overexpressed in glioma, and its overexpression is associated with poor patient survival (Jiao et al., 2018). SMAD6 correlated with poor patient survival among non-small cell lung cancer, and its knockdown inhibited cell proliferation and increased apoptosis in the lung cancer cell line (Jeon et al., 2008). Our study indicated that overexpression of SMAD6 is related to worse patient prognosis in CRC.

Our study identified that upregulated PTRH1 and UPF3B correlated with worse prognosis of CRC patients. However, we only found a few studies about these two genes in CRC and other cancers. UPF3B, a member of the UP-frameshift proteins, mediates nonsense-mediated mRNA decay (Raimondeau et al., 2018). UP-frameshift proteins include UPF1, UPF2, UPF3A, and UPF3B (Raimondeau et al., 2018). Although UPF3B is less well-studied, we found several studies about additional UP-frameshift proteins in cancers. UPF1 regulates tumor progression via diverse mechanisms across different kinds of cancers, including CRC (Bordonaro and Lazarova, 2019), hepatocellular carcinoma (Chang et al., 2016; Zhang et al., 2017), pancreatic adenosquamous carcinoma (Liu et al., 2014), glioblastoma (Shao et al., 2019), and endometrial carcinoma (Xing et al., 2020). UPF3A partially contributes to the effect of calcium homeostasis endoplasmic reticulum protein (CHERP) in promoting tumorigenesis in CRC (Wang Q. et al., 2019). UPF3B and other UP-frameshift proteins can be interacted (Raimondeau et al., 2018). These data, combined with results from our study, suggest that UPF3B regulates tumor progression of CRC and may represent a potential prognostic biomarker for CRC patients. However, the mechanism of its effect in CRC requires further study.

Although our study indicates that RBPs prominently contribute to the prognosis of CRC patients, several limitations need to be pointed out. First, the clinical cohort contains fewer patients, which may lead to deviation. Additionally, the mechanisms of how these RBPs regulate the progression of CRC require further exploration.

## Conclusion

In conclusion, we performed a comprehensive bioinformatics analysis of RBPs and identified several potential prognostic RBPs in CRC. The prognostic risk score model, including four RBPs, is an independent prognostic factor for CRC. These four RBPs are involved in tumorigenesis, progression, and prognosis of CRC. RBPs represent an alternative strategy to interfere with tumor progression and predict the prognosis of CRC patients in the future.

## Data Availability Statement

All datasets presented in this study are included in the article/Supplementary Material.

## Ethics Statement

The studies involving human participants were reviewed and approved by the Ethics Committee of Peking University People’s Hospital. The patients/participants provided their written informed consent to participate in this study.

## Author Contributions

ZZ, LW, SW, and ZS designed the study. ZZ, LW, QW, MZ, and BW analyzed the data. ZZ and LW wrote the manuscript. KJ, YY, SW, and ZS reviewed and edited the manuscript. All authors read and approved the final manuscript.

## Conflict of Interest

The authors declare that the research was conducted in the absence of any commercial or financial relationships that could be construed as a potential conflict of interest.

## Footnotes

1 https://portal.gdc.cancer.gov/

2 https://www.ncbi.nlm.nih.gov/geo/query/acc.cgi?acc=GSE29623

3 https://cistrome.shinyapps.io/timer/

4 http://www.proteinatlas.org

5 http://www.cbioportal.org/

## Supplementary Material

Supplementary Figure 1 Test of four RBPs-related gene model in the GSE29623 cohort. **(A)** Overall survival curve for high-risk and low-risk groups in the GSE29623 cohort. **(B–D)** Heat map of mRNA expression **(B)**, distribution of risk score **(C)**, and survival status **(D)** of patients in high-risk and low-risk groups in the GSE29623 cohort, respectively.
