# Supplementary figures and images for: Molecular Characterization and Clinical Relevance of RNA Binding Proteins in Colorectal Cancer
Source: Front Genet. 2020 Oct 16;11:580149. doi: 10.3389/fgene.2020.580149 (PMC7597397; doi:10.3389/fgene.2020.580149)

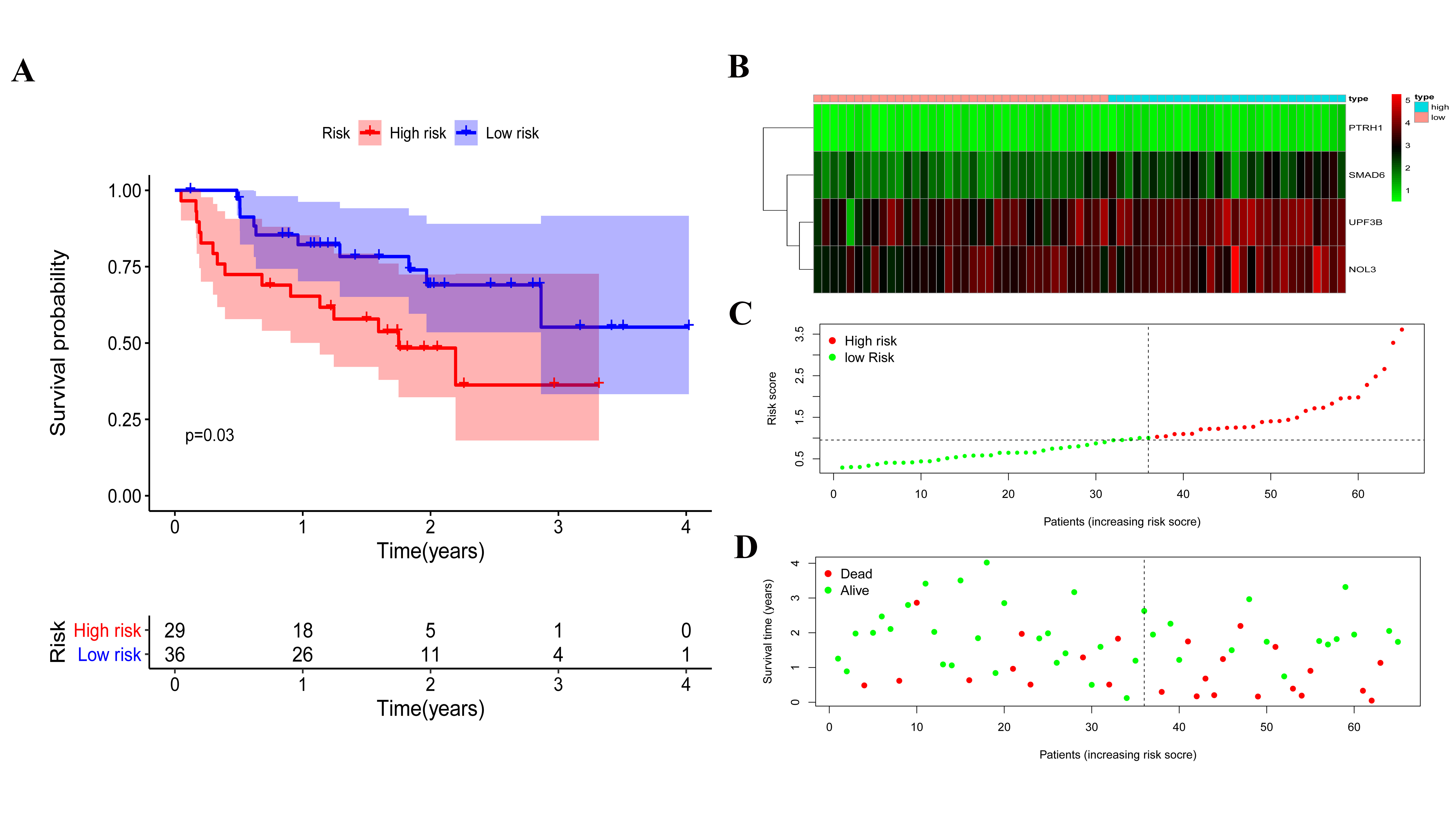

Supplement: Supplementary Figure 1 — Test of four RBPs-related gene model in the GSE29623 cohort. (A) Overall survival curve for high-risk and low-risk groups in the GSE29623 cohort. (B–D) Heat map of mRNA expression (B), distribution of risk score (C), and survival status (D) of patients in high-risk and low-risk groups in the GSE29623 cohort, respectively. [file Image_1.JPEG]
